# Type III NRG-1 plays a regulatory role in the regeneration process of nerves from the beginning of transplantation

**DOI:** 10.1186/s13018-023-04191-9

**Published:** 2023-09-20

**Authors:** Jun-Ning Wang, Sai He, Wei-xia Yang, Yao Lu, Kun Li, Yu-Min Zhang, Ya-Kang Wang

**Affiliations:** 1 Department of Orthopedics, Honghui Hospital, Xi’an Jiao Tong University, Xi’an, 710054 People’s Republic of China; 2 Department of Breast Cancer, Shaanxi Provincial Cancer Hospital, Xi’an, Shaanxi China; 3 Department of Pathology, Genertec Universal Xihang Hospital (Xi’an) Co., Ltd., Xi’an, 710021 People’s Republic of China; 4 Department of Respiratory, Honghui Hospital, Xi’an Jiao Tong University, Xi’an, 710054 People’s Republic of China

**Keywords:** Neuregulin-1 (NRG-1), Myelin sheath, Regeneration, Sciatic functional index, Antisense oligonucleotide, Autologous nerve transplantation, Sprague–Dawley rats

## Abstract

The present study investigated the effect of type III Neuregulin-1 (NRG-1) on changes in the myelin sheath and the recovery of nerve function during the regeneration process following autologous nerve transplantation. Seventy-two Sprague–Dawley rats were divided into a Blank, Model and (antisense oligonucleotide, ASON) group. The Model and ASON groups of SD rats were subjected to autologous nerve transplantation, and the Blank group only had the sciatic nerve exposed. The Model and ASON groups were given local injections of 2 ml PBS buffer solution and 2 ml ASON of Type III NRG-1, respectively, the NRG-1 type III was inhibited by ASON. Changes in the sciatic nerve functional index (SFI) and conduction velocities were observed at different 6 time points. Regeneration of the myelin sheath was observed using transmission electron microscopy. Type III NRG-1 protein was detected using Western blotting and immunohistochemistry, and NRG-1 mRNA was detected using PCR. The SFI of the ASON group was lower than the Model group after transplantation. The conduction velocities of the ASON group on the 14th and 21st days after autologous nerve transplantation were lower than the Model group (*P* < 0.01). The protein and mRNA expression of type III NRG-1 in the ASON group was lower than the Model group at all 6 time points. The area of medullated nerve fibres was significantly different between the ASON group and the Model group on the 3rd day (*P* < 0.05), as was the number of medullated nerve fibres per unit area (*P* < 0.01). The diameter of axons was obviously different between the two groups (*P* < 0.01). Type III NRG-1 played an important regulatory role in the regeneration process of the nerve from the beginning of transplantation to the 28th day.

**Supplementary Information:**

The online version contains supplementary material available at 10.1186/s13018-023-04191-9.

## Introduction

During the process of peripheral nerve development, type III NRG-1 plays a key regulatory role in the development of the whole Schwann cell pedigree in combination with ERBB2 and EerbB 3 [1]. Type III NRG-1 is cleaved by multiple sheddases and processed by three different I-Clips [2], and it is primarily expressed in the sensory and motor neurons of humans and mammals [3]. Type III NRG-1 plays a regulatory role in the development and formation of neural crest cells. It also regulates the development of the precursor cells of Schwann cells into mature Schwann cells and the number of Schwann cells formed. Type III NRG-1 is also found in bone and muscle tissue, where it regulates the development and formation of muscles [4]. The protein expression levels of type III NRG-1 determine the fate and result of myelinization of axons, plays a decisive role in the regulation of the thickness of the myelin sheath and affects changes in nerve conduction velocity.

After peripheral nerve injury, Schwann cells play an important role in nerve repair. Under the regulation of the surrounding microenvironment, cells and molecules, Schwann cells gradually surround axons and form medullated nerve fibres. Wallerian degeneration destroys the connection between axons and Schwann cells and affects the entire process of repair [5]. After peripheral nerve injury, the EerbB 2 receptor has no decisive effect on the myelination process of Schwann cells, but the redifferentiation of Schwann cells and the remyelination of axons primarily rely on the regulation of NRG-1.

However, whether type III NRG-1 plays the same role in autologous nerve transplantation in adult rats and affects changes in the myelin sheath and the recovery of nerve function after transplantation is not known. Therefore, the current study established a model of autologous nerve transplantation composed of type III NRG-1 antisense oligonucleotide that was locally injected into the incision of SD rats. Changes in SFI and motor nerve conduction velocity (MNCV) were observed, and changes in the myelin sheath were analysed under transmission electron microscopy. The mRNA and protein expression of type III NRG-1 were measured using PCR and Western blot, respectively, and the role of type III NRG-1 in the regeneration process of autologous nerve transplantation in rats was analysed.

## Methods

### Preparation of an animal model of autologous nerve transplantation

A total of 72 healthy male Sprague–Dawley (SD) rats (Housing condition: the temperature is between 18 and 26 degrees Celsius and the relative humidity is between 40 and 70%. In general, the temperature in the rat enclosure is 1–2 times higher than the ambient temperature, and the humidity is 10% higher. The noise level was below 85 dB and the ammonia concentration was below 20 ppm. Ventilation lasted 8–12 hs), of clean grade, weighing 250–300 g, 6–8 weeks old were provided by the animal experimental centre of Xi’an Jiaotong University and randomly divided into a Blank group, Model (Buffer Solution) group and antisense oligonucleotide NRG-1 type III (ASON) group. There were 24 rats in each group. Six time points were set on the 3rd, 7th, 14th, 21st, 28th and 35th days after surgery, and 4 rats in each group were tested at each time point.

**Fig. 1:**
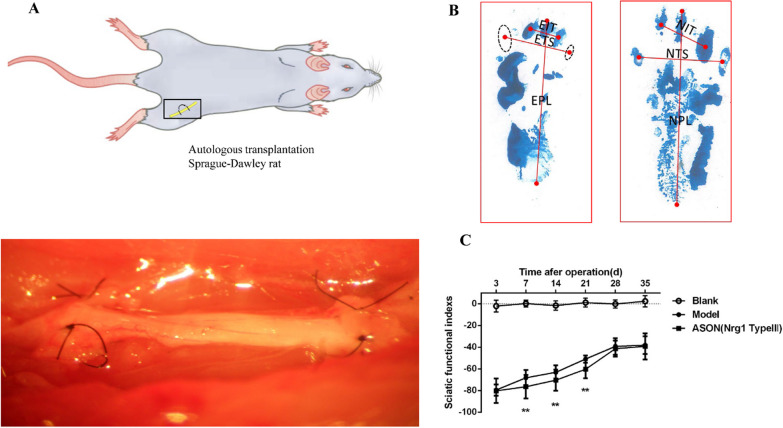
**A** The surgical procedure for autologous sciatic nerve grafting in the Model and ASON groups: The rat hind limb model was opened and the sciatic nerve exposed, rotated 180° and repaired. Sutured with two 11–0 epineural sutures under a microscope at 10× magnification at the bottom; **B** The SFI acquisition and measurement methods: Surgical side footprint (left hind limb), the dotted ellipse is the footprint of the rat that did not fully step on the centre of the ellipse is taken as the starting point for measurement; the contralateral non-surgical footprint (right hind limb);** C** The change in sciatic nerve functional index between the 3rd and 35th day after surgery; **P < 0.01 (two-way ANOVA analysis), which is the comparison between each time point of the ASON group and the model group

The Model group and ASON group were subjected to autologous nerve transplantation, and only the sciatic nerve was exposed in the Blank group. The Blank group, Model group and ASON group received an intraperitoneal injection of 1% sodium pentobarbital (40 mg/kg) for anaesthesia. The skin was prepared and disinfected. A sideling incision of approximately 1.5 cm was made at the lower margin of the left ischial tuberosity, and the sciatic nerve was identified after muscle separation. Blunt dissection was performed, and 1 cm of the sciatic nerve was incised on the lower margin of the piriformis. After 180° of inversion, epineurial suturing was performed using an 11–0 nerve anastomotic suture under a ten-fold magnification microscope (Fig. 1A)[6]. After the surgery, the sarcolemma and skin were sutured successively, and local muscle instillation of 800,000 units of penicillin was performed to diminish inflammation. The rats were housed in separate cages. Rats in the Blank group underwent sciatic nerve exposure without transplantation. Changes in rat footprints were observed at different time points, and the sciatic nerve index (SFI, the sciatic nerve function index is the same meaning as the sciatic nerve index) was calculated. Electrophysiological detection of motor neuron conduction velocity was performed. Changes in nerve terminal myelination regeneration after surgery were observed using transmission electron microscopy. Changes in type III NRG-1 protein were detected using Western blotting, and changes in type III NRG-1 mRNA were detected using RT-PCR transplantation. The expression level of neuregulin-1 type III protein was observed using immunohistochemistry. The remaining procedures were the same as the Blank group.

According to the SD rat type III NRG-1 mRNA sequence (NCBI AF 194438), computer software was used to design oligonucleotides targeting the type III NRG-1 gene, and the antisense oligonucleotide sequence was 5′-rGrGrArArCrUrCrArGrCrCrArCrArArArArCrArATT-3′, which was

synthesized by Nanjing KingsRui Biological Co., Ltd (Nanjing, China). The total synthesis was 2 OD260 and 31.79 μg/OD260. The oligos were refrigerated at low temperature, and diluted with sterile PBS buffer at a ratio of 1 μg:1 mL. (1) The Blank group received a local injection of 2 ml saline solution to the wound (between the lower margin of the ischial tuberosity and the wound) immediately after surgery and on the third day after surgery. (2) The Model group received a local injection of 2 ml PBS buffer solution to the wound immediately after the transplantation and on the third day after surgery. (3) The ASON group received a local injection of 2 ml ASON (NRG-1 Type III, 2 μg) to the wound immediately after the transplantation and on the third day after surgery. NRG1, specifically the membrane-bound type III isoform, is the signal responsible for activating this transcription factor. Since NRG-1 type III was generated in the period between the 1st and the 7th day after auto-autologous nerve transplantation, we inject the ASON to inhibit the NRG-1 type III [7, 8]*.*

### Footprint collection and calculation of the SFI after transplantation

On the 3rd, 7th, 14th, 21st, 28th and 35th days after surgery, 4 rats from each group were selected, and the soles of their hind feet were coated with blue ink. The rats were placed on a track with white paper (length of 75 cm × width of 10 cm × height of 15 cm), and the bilateral footprints of the rats were recorded. At least 5 complete sets of footprints were recorded for each experimental animal. The SFIs were measured and calculated using electronic scanning and PRISM6.0 software. The formula was SFI = (-38.3 × (EPL-NPL)/NPL) + (109.5 × (ETS + NTS)/NTS) + (13.3 × (EIT-NIT)/NIT)-8.8, where EPL and NPL represent the distance between the heel and the tip of the third toe; ETS and NTS represent the distance between the first toe and the fifth toe; and EIT and NIT represent the distance between the second toe and the fourth toe. E (experimental limb) represents the footprint on the operated side (left hind foot), and N (non-operated limb) represents the contralateral non-operated footprint (right hind foot, Fig. 1B) [9].

After the measurement of footprints, intraperitoneal anaesthesia was administered to the rats in each group on the 3rd, 7th, 14th, 21st, 28th and 35th days after surgery.

### Measurement of the conduction velocity of motor neurons

On the 3rd, 7th, 14th, 21st, 28th and 35th days after surgery, 4 rats from each group were selected. Rats were anesthetized and placed in the prone position. The system was started, and the experimental item was selected: action potential conduction speed of the nerve stem. The measurement length was set as 5 CM. The stimulus electrodes were connected: two needle electrodes were inserted into the sciatic nerve incision S1 (2 mm bipolar spacing). The red alligator clip of the stimulus electrode (positive electrode) of BL 420 was placed at the proximal end, and the black clip was placed at the distal end (negative electrode). The recording electrodes were connected: the recording electrode was connected to S2 (outside the ankle joint). The red alligator clip (positive electrode) was connected to the input channel (channel 1) and placed at the proximal end, and the white clip was placed at the distal end (negative electrode).

The reference electrode was connected: the black alligator clip (ground wire) connected to the input channel was connected to the proximal end 1 cm away from S2 (or the tail of the rat). Square wave stimulation: a single stimulus was applied, with a 5 ms delay, 5 ms bandwidth, 50 Hz frequency, and 10 V record conduction time. The onset of stimulation to the appearance of action potential and *t*_1_ was recorded.

The amplitude of the action potential wave was recorded. There were two peaks (the difference between the highest peak and the lowest peak of the inverted wave), which were measured 8 times, and the average value was taken. The distance between the recording electrode and the stimulating electrode was measured. The left lower limb of the rat was straightened to 45° from the spine, and the distance between the sciatic nerve and the recording electrode was measured and recorded as *L*_1_. The stimulating electrode at S2, the recording electrode at R, and the reference electrode at 1 cm proximal to R were connected in the same manner. The conduction time (*t*_2_), amplitude of the action potential, and distance between the recording electrode and stimulating electrode (*L*_2_) were recorded in the same manner. Motor nerve conduction velocity (MNCV) was calculated: $MNCV = \frac{{L_{1} - L_{2} }}{{t_{1} - t_{2} }}$. The experimental method was shown in Additional file 1: Fig. S1.

The MNCV was recorded, and the experimental results were saved for statistical analyses. The nerve conduction velocity was measured, and rats in each group received intraperitoneal anaesthesia of 1% sodium pentobarbital (40 mg/kg) on the 3rd, 7th, 14th, 21st, 28th, and 35th days after surgery. The intermediate segment of the operated transplanted sciatic nerve of the ASON and Model groups and the corresponding nerve segment of the blank group were cut into four 0.25-cm long sections. One section was placed into 0.1 M alkaline phosphate buffer to prepare the specimen for electron microscopic observation. The other sections were placed at − 80° for Western blotting, RT-PCR and immunohistochemistry.

### Regeneration of the myelin sheath was observed using transmission electron microscopy

The sciatic nerve samples were prepared, and 1 mm^3^ of tissue was cut from each group, fixed for 2–4 h in 3% glutaraldehyde and 0.1 M phosphate buffer, rinsed with 0.1 M phosphate washout fluid and fixed with 1% osmium tetroxide. The samples were dehydrated with 50%, 70%, 90% and 100% acetone successively. Dehydrated samples were soaked in 100% acetone at room temperature, embedded in solution (1:1) and solidified in the oven. The samples were sectioned ultrathin slices (50 nm), and double staining with 3% uranyl acetate-lead citrate was performed. The following indicators were observed and recorded during transmission electron microscopy(JEM-1400, Japan): ① area of regenerated nerve; ② number of nerve fibres per unit area (number/339.75 μm^2^); ③ average diameter of the medullated nerve fibres; and ④ degree of myelination of medullated nerve fibres (G-ratio, the ratio of the diameter of the axon to the diameter of the nerve fibres) [10, 11].

### Variable expression of type III NRG-1 was detected using Western blotting

The prepared sciatic nerve was minced, ground, cracked and placed in a 1.5 ml centrifuge tube, 10 µl of phenylmethanesulfonyl (100 mM) was added to 1 ml of lysate in a homogeniser, shaken and placed on ice. After lysis for 30 min, the supernatant was centrifuged at 4 °C for 25 min at 12,000 rpm. The supernatant was then collected and placed into a centrifuge tube. Tissue protein was extracted (Beijing Solarbio).

Science & Technology Co., Ltd.). The protein content was determined using bicinchoninic acid (Thermo Fisher Scientific, Inc). Proteins (20 μg/lane) were separated using 10% SDS‑PAGE and were then transferred onto a PVDF membrane (Millipore Sigma). The membranes were blocked with 5% skimmed milk in TBST containing 0.05% Tween‑20 for 1 h at room temperature and incubated with SDS–polyacrylamide gel electrophoresis and transferred to a polyvinylidene fluoride filter membrane. The membrane was incubated with primary antibody (HRG-C19 goat polyclonal anti-Nrg-1 (Santa Cruz Biotechnology, Santa Cruz, CA; 1:1000)), overnight at 4 °C, and secondary antibodies (RG-16 mouse monoclonal anti-rabbit Ig 1:3000 (Sigma)) conjugated with peroxidase for 1 h at room temperature, added and a chemiluminescence reaction was performed (Clarity™ Western ECL Substrate; Bio‑Rad Laboratories, Inc). GAPDH (R&D systems, Abingdon, UK; 1:500,10 ng per lane) was used as the control for normalization. Protein bands were developed and fixed, and the film was scanned or photographed. The molecular weight and net optical density of the object band were analyzed using ImageJ software (Version 1.49, NIH).

### Variable expression of type III NRG-1 was detected using PCR

The tissue was ground, weighed and loaded into a centrifuge tube. TRIzol reagent was added, and 0.2 ml of chloroform and isopropanol were successively applied to extract the total RNA. DEPC-treated water was added for dilution, and the sample concentration was determined using UV spectrophotometry. OD 260 and OD 280 was determined, and cDNA was synthesized via reverse transcription.

The following primer sequences of EGF-like domain NRG1-type III were used:

Forward:5′-3′: GACCCCTGAGGTGAGAACAC;

Reverse: 5′-3′: CCCCCATTCACACAGAAAGT [12].

The GAPDH was used:

Forward:5′-3′: GACAACTTTGGCATCGTGGA.

Reverse: 5′-3′: ATGCAGGGATGATGTTCTGG.

A real-time fluorescence quantitative PCR system was established, and thermal cycling was performed with the following amplification conditions: 95 °C for 10 min followed by 40 cycles of 30 s at 95 °C and 2 min at 59 °C [12, 13]. The data were exported when the reaction ended, and the amount of target cDNA was determined by the number of cycle times (Ct) of threshold amplification as calculated by ABI Sequence Detection System software. The difference between the Ct value of the target gene and the internal reference GAPDH was the relative Ct value. The relative DNA content was calculated by the following formula: average relative content = 2^−average ΔΔCT^.

### Immunohistochemistry

The sciatic nerve was prepared at various time points, post-fixed, and sectioned. Sections were subjected to immunofluorescent staining following a standard procedure. Briefly, sections were incubated with 0.3% (v/v) Triton-X for 30 min then blocked in 10% horse serum (Biosharp BL1098A) for 2 h at 4 °C. The sections were incubated with a rabbit anti-neuregulin-1 type III polyclonal antibody (1:400; Abcam Inc. Cat. no: abs135646) and mouse anti-NF200 monoclonal antibody (1:200; Sigma. Cat. no: N5389) at 4 °C for 24 h. The primary antibodies were probed with indocarbocyanine-conjugated goat anti-rabbit monoclonal antibodies overnight at 4 °C (1:1,000; Abcam Inc. A28277) or fluorescein-conjugated goat anti-mouse monoclonal antibody for 1 h at 4 °C (1:1,000; Abcam Inc. A32032), and DAPI (10 μg/mL; Sigma), a fluorescent nuclear dye. All of the incubation steps, except the overnight incubation, were performed at room temperature. Samples were rinsed three times in PBS (pH 7.4) between each step. Sections were examined under a laser confocal microscope (FV1000; Olympus, Tokyo, Japan). All images were taken under the same conditions and parameters. Red fluorescence intensity represented the expression level of neuregulin-1 type III protein. The rats' death verification was conducted by spinal dislocation at the end of the experiment under intraperitoneal anaesthesia.

### Statistical methods

The IBM SPSS Statistics 20 and Prism 16 software packages were used for analyses. The data are expressed as the means ± standard deviation. The SFI, MNCV, changes of protein expression of and mRNA of type III NRG-1, measurement of axon and myelin were analyzed by two-way ANOVA repeated measure 6 times between the ASON (NRG-1 type III) group and the Model group, followed by Tukey’s post hoc test. *P* < 0.05 was considered statistically significant.

## Results

### Analysis of footprint results

The footprints of rats were recorded on the 3rd, 7th, 14th, 21st, 28th and 35th days after nerve transplantation, and the sciatic functional index was calculated to evaluate the motor function recovery of rats after nerve injury and the inhibition of type III NRG-1. On the 7th, 14th and 21st days after surgery, the SFI of the ASON group was lower than the Model group, and the difference was statistically significant (*P* < 0.01). These results suggest that the reduction in type III NRG-1 slowed the early recovery of sciatic nerve function in rats after autologous nerve transplantation (Fig. 1C, GraphPad Prism 6 was used to generate this image).

### Determination results of motor neuron velocity

**Fig. 2:**
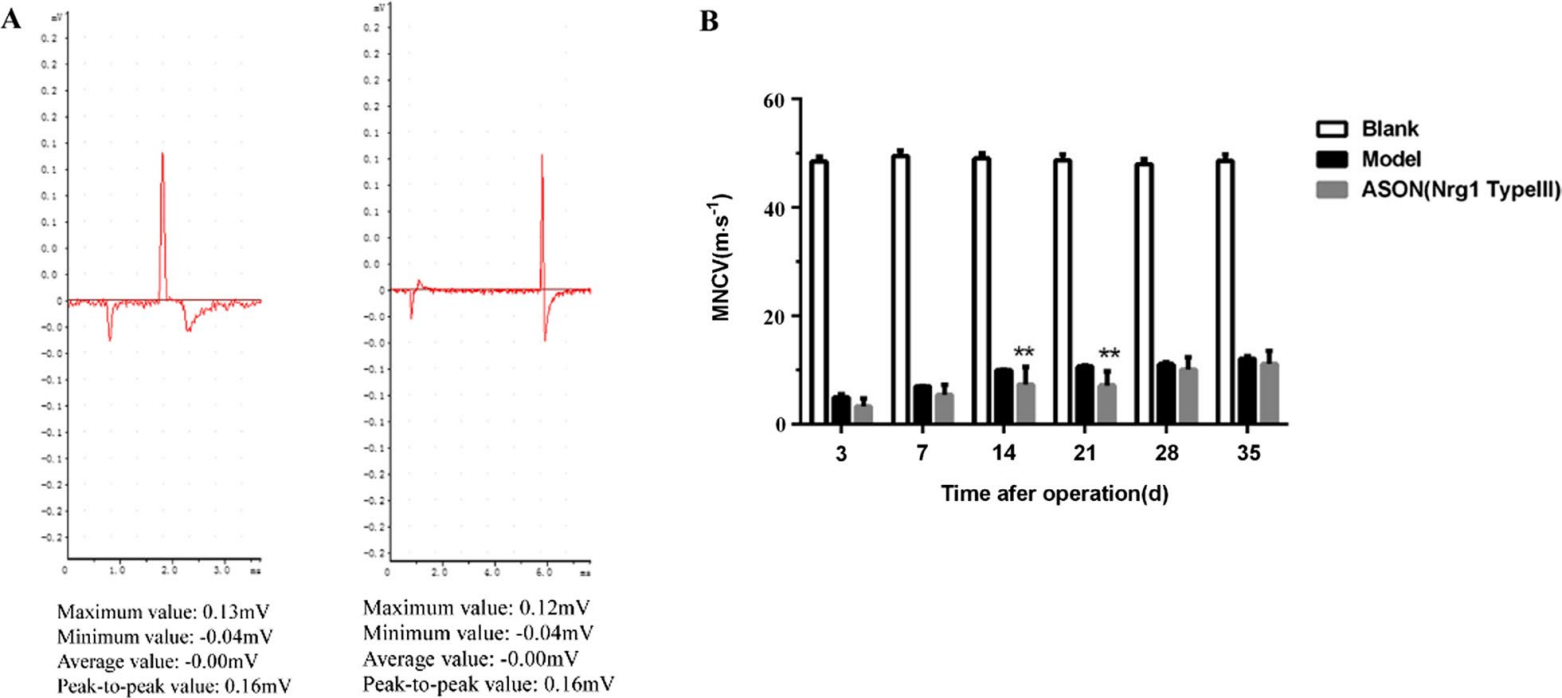
**A** Schematic diagram of sciatic nerve evoked potential in SD rats: Left: Model group; Right: ASON group. **B** Bar chart of MNCV after autologous nerve transplantation in rats: ** ASON group was compared with Model group, **P < 0.01(two-way ANOVA analysis)

On the 3rd, 7th, 14th, 21st, 28th and 35th days after autologous nerve transplantation, the changes in MNCV in the sciatic nerve of rats were detected, as shown in Fig. 2A. The evoked potential maps of MNCV of the Model group and the ASON group were directly measured in vitro using the BL-420 biofunctional system.

The conduction velocities of the ASON group on the 14th and 21st days after autologous nerve transplantation were lower than the Model group, and there were statistically significant differences (*P* < 0.01 at both time points, Fig. 2B). No significant difference was found at other time points. These results suggested that inhibition of the expression of type III NRG-1 slowed the motor neuron conduction velocity of the sciatic nerve of rats at 14–21 post-surgery.

### Western blot

**Fig. 3:**
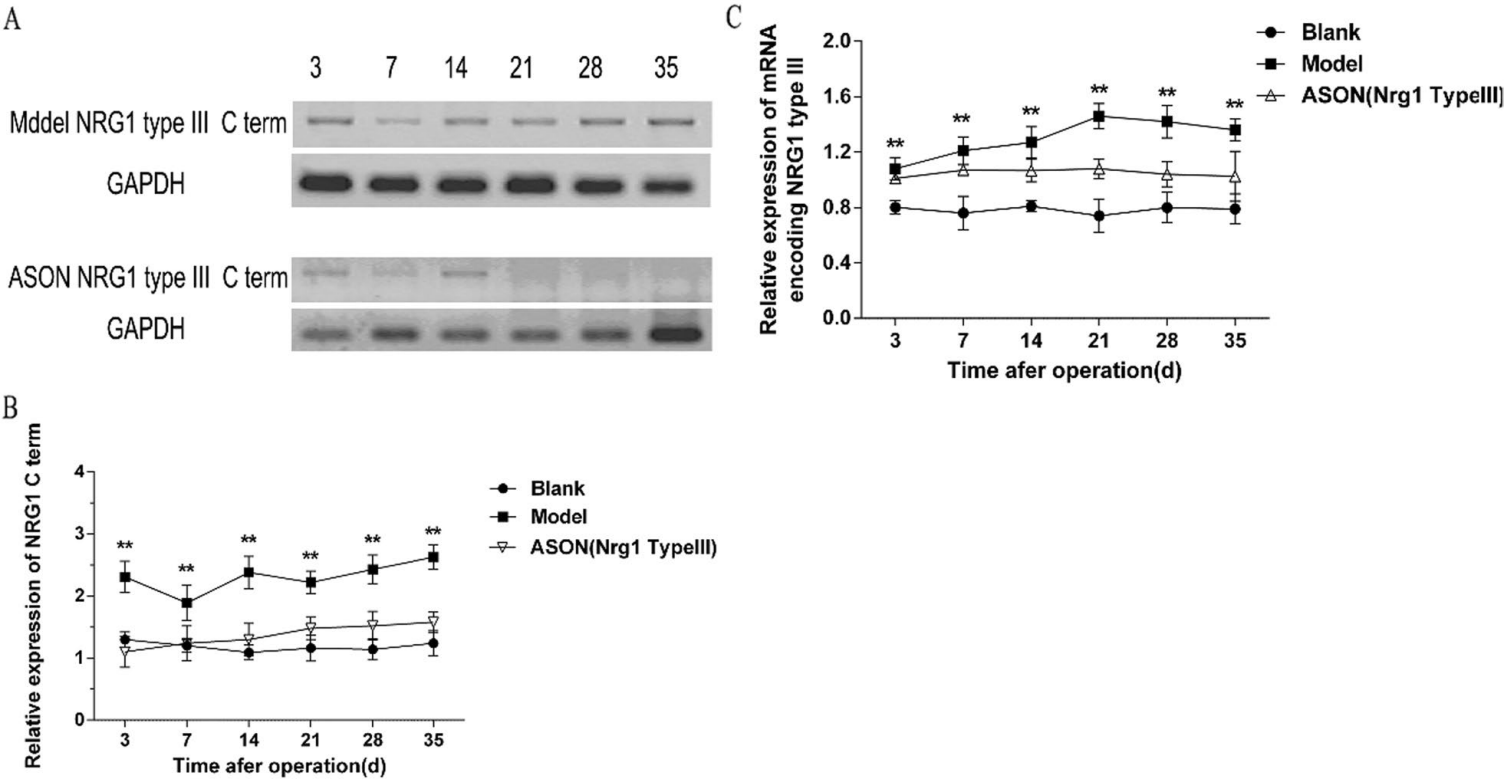
**A**–**B** Protein expression of NRG-1 type III in the model and ASON groups was detected by Western blot between 3 and 35 days after surgery; ** represents *P* < 0.01(two-way ANOVA analysis), showing that the differences between the model and ASON (NRG-1 type III) groups were statistically significant at all time points. **C** After autologous nerve transplantation of sciatic nerve in SD rats, antisense oligonucleotide was locally injected to inhibit the expression of type III NRG-1, and the changes of mRNA of type III NRG-1 were detected by RT-PCR between 3rd day and 35th day after surgery; ** represents *P* < 0.01(two-way ANOVA analysis), which means the comparison between the ASON (NRG-1 type III) group and the Model group at each time point with statistically significant difference

The Western blot results showed that the expression of type III NRG-1 in the ASON group was significantly reduced compared to the Model group at 6 time points between the 3rd day and 35^th^ day after autologous nerve transplantation in SD rats. These results demonstrated that local injection of antisense oligonucleotide through the incision of rats effectively inhibited the protein expression of type III NRG-1 (Fig. 3A, B).

### RT-PCR results

The RT-PCR results showed that local injection of antisense oligonucleotides after sciatic nerve autologous transplantation inhibited the expression of type III NRG-1. The mRNA expression of type III NRG-1 in the ASON group was decreased on the 3rd, 7th, 14th, 21st, 28th and 35th days after nerve transplantation compared to the Model group (*P* < 0.01, respectively; Fig. 3C). These results showed that the expression of type III NRG-1 in the ASON group was effectively inhibited.

### Observational results of transmission electron microscope

**Fig. 4:**
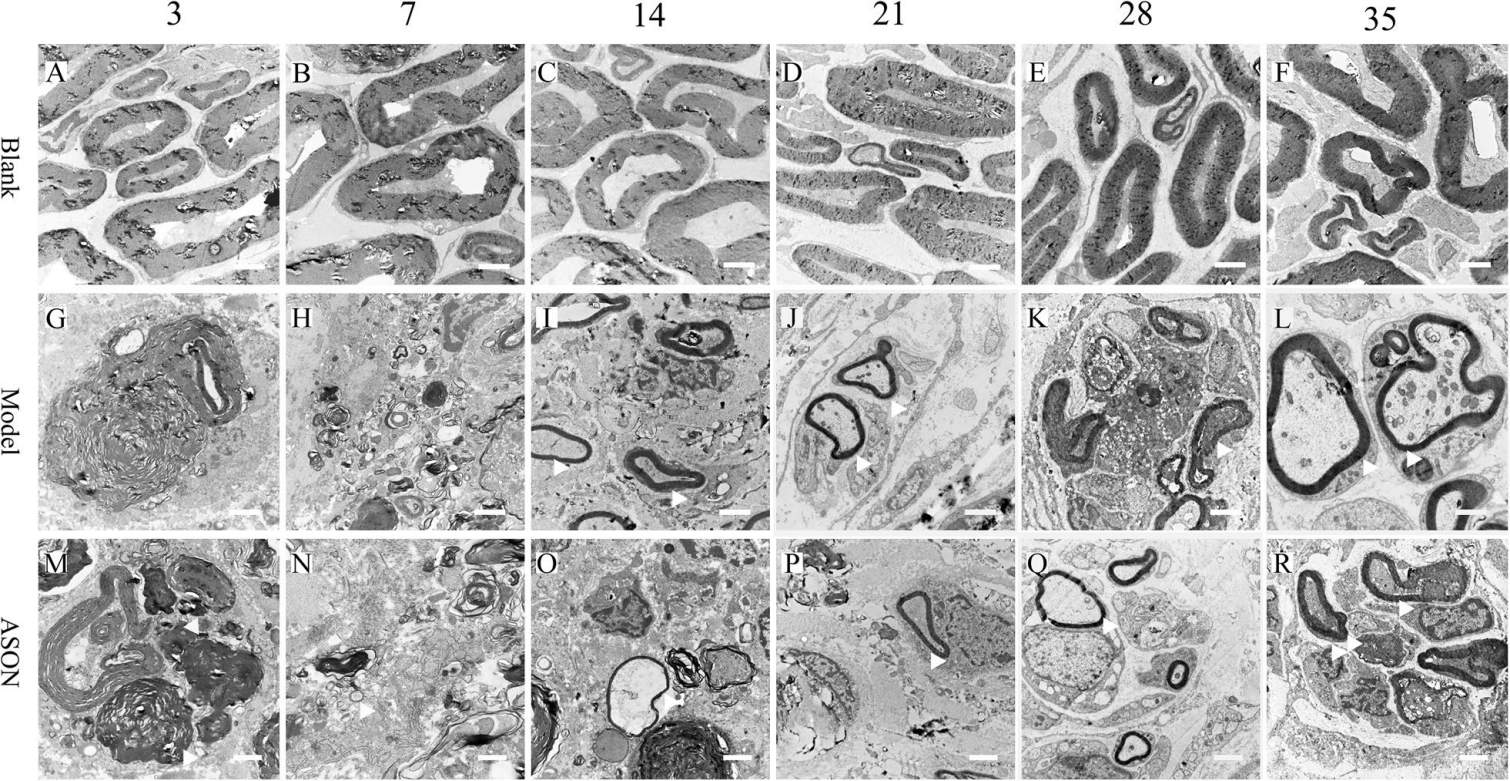
Results of the observation by transmission electron microscope: all scales were 2 microns, 8000 times magnification. Blank group: **A**–**F**, Model group: **G**–**L**, ASON group: **M**–**R**. The white triangle is marked for the site of myelin. The Blank, Model and ASON is the group information on the left. The 3, 7, 14, 21, 28 and 35 are the specific time points on the top of the figure

Morphological observations using transmission electron microscopy (all scales are 2 microns, 8000 times magnification): The myelin sheath of medullated nerve fibres began to crack 3 days after transplantation in the ASON group (Fig. 4M). A relatively small myelin sheath was observed on the 7th day after transplantation (Fig. 4N) The diameter of nerve fibres became larger on the 14th and 21st days after surgery, but the myelin sheath in the ASON group was thinner than the Model group (Fig. 4I, J, O, P). There was no significant difference between the morphology of the myelin sheaths in the ASON and Model groups (Fig. 4K, L,Q, R).

**Fig. 5:**
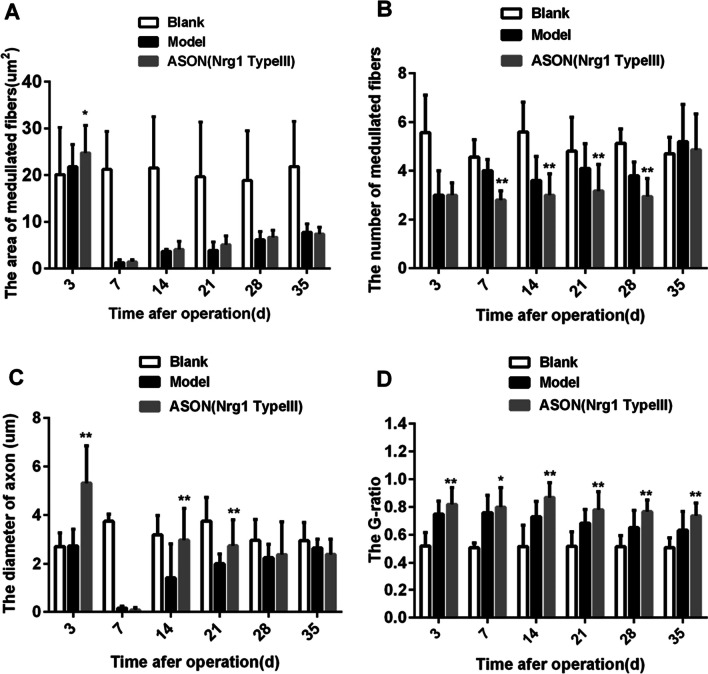
Bar chart of analysis of electron microscope results: **A** area of medullated nerve fibres (μm^2^); **B** number of medullated nerve fibres per unit area (number /339.75μm^2^); **C** diameter of the axon (μm); **D** G–ratio; At least 20 axons of each specimen were measured for statistical analysis: *P < 0.05 ASON VS Model, the difference was statistically significant; ** *P* < 0.01 ASON VS Model, the difference was statistically significant (two-way ANOVA analysis)

Results of measurement and analysis. The area of medullated nerve fibres (μm^2^): There was a significant difference between the ASON group and the Model group on the 3rd day (*P* < 0.05, Fig. 5A), but no significant difference was observed in the later stages. For the number of medullated nerve fibres per unit area, there were significant differences between the ASON group and the Model group on the 7th, 14th, 21st and 28th days (*P* < 0.01, Fig. 5B). These results indicated that the number of nerve fibres per unit area in the ASON group was less than the Model group between the 7th day and 28th days. Axon diameter: Significant differences between the ASON group and the Model group were found on the 3rd, 14th and 21st days (*P* < 0.01, Fig. 5C). The G-ratio at all time points was significantly higher in the ASON group than the Model group (*P* < 0.05, Fig. 5D).

### Immunofluorescent staining

**Fig. 6:**
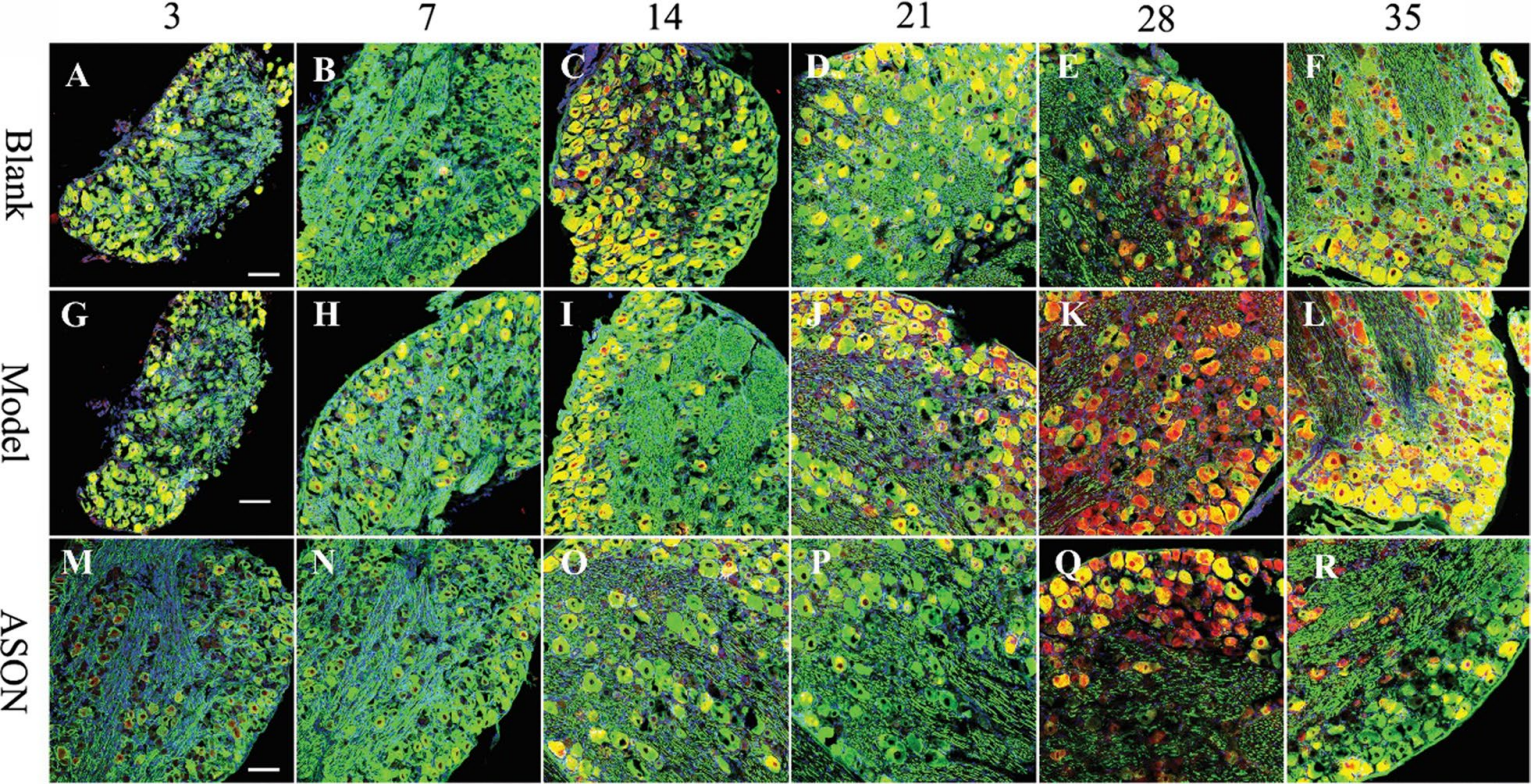
Immunofluorescence staining of NRG1 type III isolated from sciatic nerve at different time points of transplantation under laser confocal microscopy. NRG-1 type III expression was labelled with anti-NRG1 type III (red). Neurofilament expression was labelled with NF-200 (green) and nuclei were labelled with DAPI (blue). Sections were stained using the same staining procedure and all images were taken under the same conditions and parameters. Scale bars: 100 μm

To further confirm the expression pattern of neuregulin-1 type III at different time points after sciatic nerve autologous transplantation, we used immunofluorescent staining. Neurofilament was used as a positive control, and cell nuclei were labelled with DAPI. All images were taken under the same conditions and parameters. As shown in Fig. 6, the red fluorescence intensity represents the expression level of type III NRG-1 protein, which tended to increase gradually from the 3rd to 28th days, peaked at the 28th day, then decreased at the 35th day in the Model group (Fig. 6G–L). Lower staining intensity was observed in the Blank (Fig. 6A–F) and ASON groups (Fig. 6M–R). Figure 6K shows the 28th day in the Model group, Fig. 6E shows the 28th day in the Blank group, the sciatic nerve only had been exposed. In the Model and ASON groups, the SD sciatic nerve was subjected to autologous nerve grafting, and in the ASON group, type III NRG-1 was inhibited by ASON. The immunofluorescent staining results showed similar results as the Western blotting and RT-PCR results.

## Discussion

During the process of neural development, type III NRG-1 in axons plays a key role in the proliferation, differentiation and myelination of Schwann cells. After peripheral nerve injury, axons undergo Wallerian degeneration and release type III NRG-1 simultaneously via the NRG-1/ErbB signalling pathway, which acts on Schwann cells to regulate the myelination process. Our results showed that the expression of type III NRG-1 was inhibited in a model of autologous nerve transplantation, and the SFI value of the ASON group was reduced compared to the Model group between the 7th day and 21st day after sciatic nerve transplantation. The MNCV value of the ASON group was also smaller than the Model group between the 14th day and 21st day after transplantation, which demonstrated that reduction of type III NRG-1 slowed the early recovery of nerve function in rats after autologous nerve transplantation. Based on the observation and analysis of myelin sheath using electron microscopy, we found that the area of the medullated nerve fibres (μm^2^) in the ASON group was significantly different from the Model group on 3rd day (*P* < 0.05). However, no significant difference was found at other time points. These results suggested that type III NRG-1 had certain effects on the disintegration of the myelin sheath. The number of medullated nerve fibres per unit area was significant significantly different between the ASON group and the Model group on the 7th, 14th, 21st and 28th days (*P* < 0.01), which indicated that the number of medullated nerve fibres without type III NRG-1 was decreased. Significant differences in axon diameter between the ASON group and the Model group appeared on the 3rd, 14th and 21st days (*P* < 0.05). The G-ratio for the ASON group was higher than the Model group at each time point, with a significant difference (*P* < 0.05), which indicated that reducing type III NRG-1 delayed the process of normal myelinisation.

Type III NRG-1 plays an indispensable role in the regulation of myelin sheath thickness during the survival and differentiation of Schwann cells [14]. Different NRG-1 isoforms play synergistic roles in nerve injury repair. Type II NRG-1 trend from 1sd to 28th in the following autologous nerve transplantation did not give any intervention experiment [6]. We not only use the ASON technique but also autologous nerve transplantation in our experiment. Besides, type II NRG-1 and type III NRG-1 have different functions during regeneration after autologous nerve transplantation [6]. Type II NRG-1 expression peaked between the 3rd day and 14th day after autologous nerve transplantation and is likely involved in the regulation of myelin sheath regeneration during this period. type III NRG-1 may mainly modulate the disintegration, number and process of the myelin sheath.NRG-1 type I is a trigger in nerve remyelination [15]. Our study shows type III NRG-1 had certain effects on the disintegration of the myelin sheath at the beginning of remyelination. Type I and type III NRG-1 may play a synergistic role in the disintegration and regeneration of the myelin sheath after nerve injury. The study by Kerber G et al. shows that, in the facial lesion, the type III mRNA is dramatically decreased 3d after the lesion and almost recovers within 14th day after lesion, and NRG-1 type III mRNA is decreased after axotomy [16]. The NRG-1 type III was suppressed by the ASON of NRG-1 type III in our study. There have been some in vitro and in vivo experiments that have demonstrated that NRG-1 ASON can inhibit NRG-1 [17, 18]. Michailov et al. found that Schwann cell precursor cells in type III NRG-1 gene knockout mutant mice continued to develop into normal Schwann cells and myelin, but the myelin, was flawed, myelin sheath thickness was decreased, the G-ratio was increased, and the number of axons and the nerve conduction velocity decreased during the process of development [19, 20]. Mice overexpressing type III NRG-1 showed increased myelin sheath thickness, decreased G-ratio and no significant change in the number of axons during the development process [20]. However, Stassart RM et al. found that the knockout of the EFG site of the NRG-1 gene of Schwann cells (i.e. inhibition of NRG-1 secretion of Schwann cells) using the CreloxP recombinase system produced no significant differences in the G-ratio of the myelin sheath or the distribution of axons compared with normal mice [21]. These studies suggest that type III NRG-1 plays an important role in the regulation of myelin sheath thickness during neural development. In addition to type III NRG-1 derived from Schwann cells, other NRG-1 sources may be involved in the regulation of myelin sheath thickness. The G-ratio at all time points was higher in the ASON group than the Model group in our study. This result shows that NRG-1 type III may also regulate sheath thickness in autologous nerve transplantation. On the 3rd day after transplantation, the diameter of axons and the area of medullated nerve fibres in the ASON group were obviously different from the Model group. During the initial stage of transplantation, the lack of type III NRG-1 in the ASON group slowed the disintegration of the myelin sheath, which was similar to Guertin et al., who reported that type III NRG-1 promoted the disintegration of axons, Schwann cells, and the myelin sheath during the early stages of peripheral nerve injury [22]. The following specific reasons may explain these results. Type III NRG-1 has chemotactic effects on macrophages, which devour axons, Schwann cells and disintegrated fragments.; Type III NRG-1 is also involved in the phagocytic process of macrophages and has a promoting effect [23]. There was a significant difference between the number of medullated nerve fibres per unit area and the diameter of axons between the 7th day and 28th day after transplantation. Canan S et al. [11] study showed there are no significant differences between these sampling strategies with respect to total number of myelinated nerve fibres, axon cross-sectional area and myelin sheet thicknesses of nerve fibres underwent stereological experiment of the tibial and peroneal nerves at 90 days. Therefore, type III NRG-1 is likely involved in the formation of the number of medullated nerve fibres and axon diameter between the 7th day and 28th day after surgery. Stassart et al. produced sciatic nerve extrusion injury in mice and found that the number of medullated nerve fibres in the mice lacking type III NRG-1 was less than the normal group on the 4th day after nerve injury, but the lack of type III NRG-1 and its receptor did not affect the number of Schwann cells [21, 24]. Rushton reported that the G-ratio of normal rats was approximately 0.6 [25], but the G-ratio after injury was higher than the normal group and exhibited a linear model [21]. However, the G-ratio of the type III NRG-1 knockout mice was significantly higher than the wild type in the mice with extrusion injuries [21]. The present experiment used a transplantation model, and all G-ratios of the Model group were higher than the ASON group. Although both studies used nerve injury, the experimental model and the means of intervention applied in the present study was different from the Rushton study. The emphasis of our experiment was that the axoplasmic transport of NRG-1 from the cell body of the neuron to the axon was cut off in the autologous nerve transplantation model, and NRG-1 is primarily derived from the synthesis and secretion of tissues, such as Schwann cells and surrounding muscles. Therefore, the various indicators presented were different from the results obtained in the present experiment.

Our experiment also has some limitations.NF200 was often applied to show nerve fibbers, but it could not present myelin, and Schwann cell conditions for the Immunohistochemistry of NRG-1. However, the performance of NF200 is stable, not only other research groups have used it for NRG-1 type III, our research group has always used it in the early stage [26–29].

A study using spinal nerve ligation as a model of nerve injury found that the mRNA expression level of type III NRG-1 was significantly higher than type I and II NRG-1 [12]. Further observation revealed that type III NRG-1 was initially inactive in the process of myelin sheath repair [30] and was activated by proteases, such as BACE 1 and ADAM 10, during the migration of Schwann cell precursor cells to the injured peripheral nerve [8, 31, 32]. After activation, it binds to the ERBB 2/3 receptor and stimulates the Schwann cell precursors to form Schwann cells, which gradually form myelinated nerve fibres [33–35].

Schwann cells are a type of redifferentiated cell involved in nerve repair after peripheral nerve injury and may be redifferentiated and myelinated after nerve injury [36]. However, the thickness of the regenerated axon decreases obviously [37, 38], and the injured nerve function cannot be completely recovered [39]. During the process of redifferentiation and remyelination of Schwann cells, various nerve factors and hormones regulate the process of myelination. During Wallerian degeneration, Schwann cells first form the Büngner band along the direction of the original axon, and the axon regrows along the Büngner band. After nerve injury, Schwann cells show abnormal myelination because of the lack of type III NRG-1, and overexpression leads to excessive myelination. After autologous nerve transplantation in this experiment [40], the ASON group lacked type III NRG-1, and abnormal myelination occurred, which caused a decrease in the SFI value and MNCV. Therefore, the authors concluded that type III NRG-1 played an important regulatory role in the regeneration process of the nerve from the beginning of the transplantation to the 28th day. These results highlight the great significance of accurately grasping the effective treatment time to administer effective drugs for treatment to improve the survival rate of the transplanted nerve.

### Supplementary Information

**Additional file 1**. Fig. S1 Motor nerve conduction velocity (MNCV) measurement in rats S1: sciatic notch stimulating electrode; S2: recording or stimulating electrode at the ankle; R: recording electrode at the interosseous muscle of the first toe; E: the reference electrode
