# Supplementary figures and images for: Type III NRG-1 plays a regulatory role in the regeneration process of nerves from the beginning of transplantation
Source: J Orthop Surg Res. 2023 Sep 20;18:707. doi: 10.1186/s13018-023-04191-9 (PMC10512478; doi:10.1186/s13018-023-04191-9)

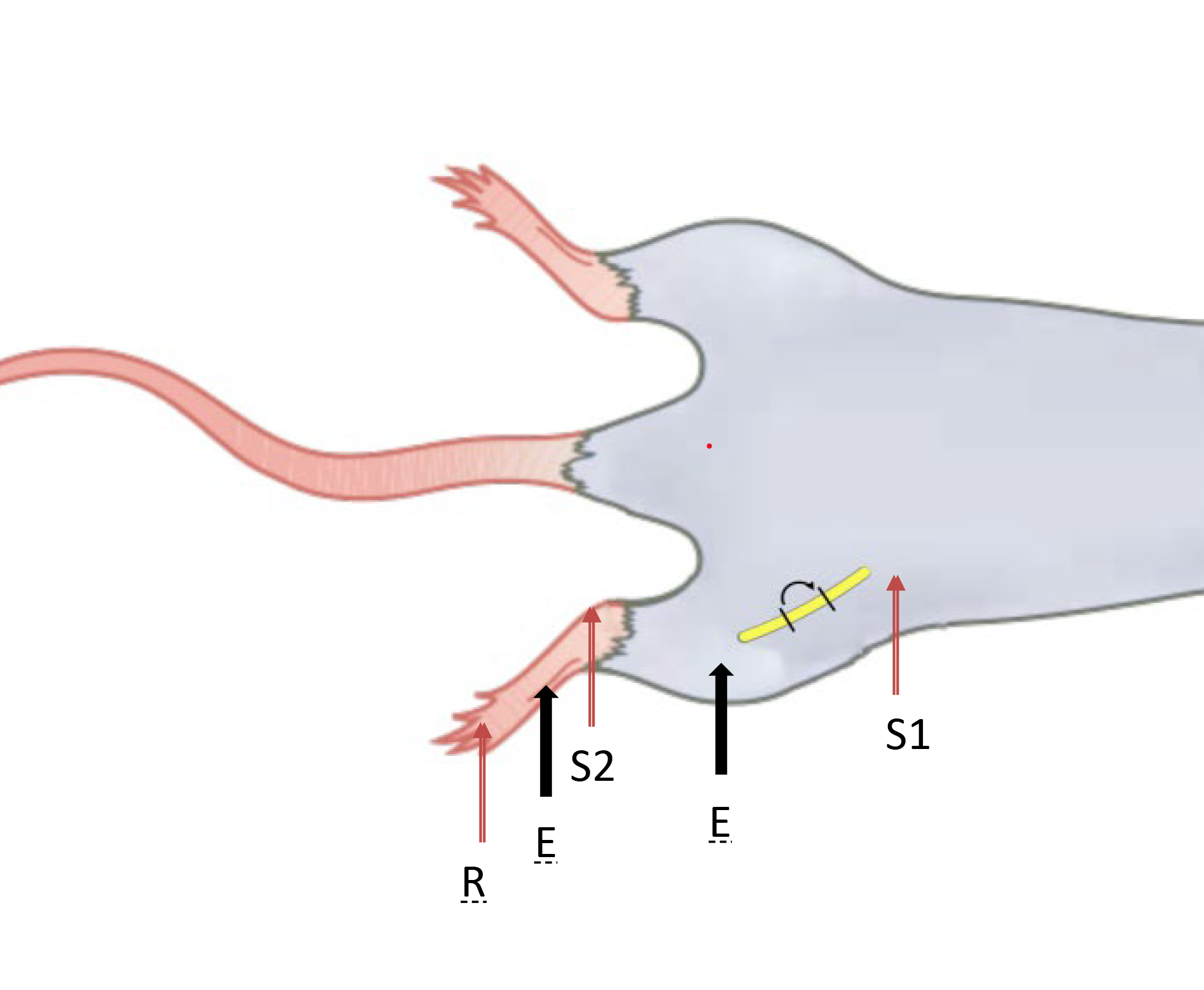

Supplement: Supplementary file 1 — Additional file 1. Fig. S1 Motor nerve conduction velocity (MNCV) measurement in rats S1: sciatic notch stimulating electrode; S2: recording or stimulating electrode at the ankle; R: recording electrode at the interosseous muscle of the first toe; E: the reference electrode [file 13018_2023_4191_MOESM1_ESM.tif]
